# Development of a Machine Learning Model for Survival Risk Stratification of Patients With Advanced Oral Cancer

**DOI:** 10.1001/jamanetworkopen.2020.11768

**Published:** 2020-08-21

**Authors:** Yi-Ju Tseng, Hsin-Yao Wang, Ting-Wei Lin, Jang-Jih Lu, Chia-Hsun Hsieh, Chun-Ta Liao

**Affiliations:** 1 Department of Laboratory Medicine, Chang Gung Memorial Hospital at Linkou, Taoyuan City, Taiwan; 2 Department of Information Management, Chang Gung University, Taoyuan City, Taiwan; 3 Healthy Aging Research Center, Chang Gung University, Taoyuan, Taiwan; 4 PhD Program in Biomedical Engineering, Chang Gung University, Taoyuan City, Taiwan; 5 School of Medicine, Chang Gung University, Taoyuan City, Taiwan; 6 Department of Medical Biotechnology and Laboratory Science, Chang Gung University, Taoyuan City, Taiwan; 7 Division of Hematology-Oncology, Department of Internal Medicine, Chang Gung Memorial Hospital at Linkou, Taoyuan City, Taiwan; 8 Division of Hematology-Oncology, Department of Internal Medicine, New Taipei Municipal TuCheng Hospital, New Taipei City, Taiwan; 9 Department of Head and Neck Oncology Group, Chang Gung Memorial Hospital at Linkou, Taoyuan, Taiwan; 10 Department of Otorhinolaryngology–Head and Neck Surgery, Chang Gung Memorial Hospital at Linkou, Taoyuan, Taiwan

## Abstract

**Importance:**

A tool for precisely stratifying postoperative patients with advanced oral cancer is crucial for the treatment plan, such as intensifying or deintensifying the regimen to improve their quality of life and prognosis.

**Objective:**

To develop and validate a machine learning–based algorithm that can provide survival risk stratification for patients with advanced oral cancer who have comprehensive clinicopathologic and genetic data.

**Design, Setting, and Participants:**

In this prognostic cohort study, the elastic net penalized Cox proportional hazards regression–based risk stratification model was developed and validated using single-center data collected between January 1, 1996, and December 31, 2011. In total, comprehensive clinicopathologic and genetic data (including clinical, pathologic, and 44 cancer-related gene variant profiles) of 334 patients with stage III or IV oral squamous cell carcinoma were used to develop and validate the algorithm in this 15-year cohort study. Data analysis was conducted between February 1, 2018, and May 6, 2020.

**Main Outcomes and Measures:**

The main outcomes were cancer-specific survival, distant metastasis–free survival, and locoregional recurrence–free survival. Model performance was compared in terms of the Akaike information criterion and the Harrell concordance index (C index).

**Results:**

Complete data were available for 334 patients (315 men; median age at onset, 48 years [interquartile range, 42-56 years]). The predictive models using comprehensive clinicopathologic and genetic data outperformed those using clinicopathologic data alone. In the groups of postoperative patients receiving adjuvant concurrent chemoradiotherapy, the models demonstrated higher classification performance than those using clinicopathologic data alone in cancer-specific survival (mean [SD] C index, 0.689 [0.050] vs 0.673 [0.051]; *P* = .02) and locoregional recurrence–free survival (mean [SD] C index, 0.693 [0.039] vs 0.678 [0.035]; *P* = .004). The classification performance in distant metastasis–free survival was not different (mean [SD] C index, 0.702 [0.056] vs 0.688 [0.048]; *P* = .09).

**Conclusions and Relevance:**

A risk stratification model using comprehensive clinicopathologic and genetic data accurately differentiated the high-risk group from the low-risk group in cancer-specific survival and locoregional recurrence–free survival for postoperative patients with advanced oral cancer. This algorithm could be used through an online calculator to provide additional personalized information for postoperative management of patients with advanced oral squamous cell carcinoma.

## Key Points

**Question:**

Can a machine learning model provide survival risk stratification for patients with advanced oral cancer who have comprehensive clinicopathologic and genetic data?

**Findings:**

In this 15-year cohort study of 334 patients, a risk stratification model using comprehensive clinicopathologic and genetic data accurately differentiated the high-risk group from the low-risk group in postoperative cancer-specific and locoregional recurrence–free survival for patients with advanced oral cancer.

**Meaning:**

The proposed model demonstrated good discrimination in stratifying patients with different risks of survival by using comprehensive clinicopathologic and genetic data, which can provide additional personalized information for postoperative management of patients with advanced oral squamous cancer.

## Teaser

This cohort study developed and validated a machine learning–based algorithm that can provide survival risk stratification for patients with advanced oral cancer who have comprehensive clinicopathologic and genetic data.

## Introduction

Current postoperative treatment of advanced oral squamous cell cancer is often a combination of chemotherapy and radiotherapy.^1^ One of the challenges for a physician is the counterpoise between treatment response and patient intolerance of toxic effects and adverse effects, including serious oral mucositis, dysphagia, speech impairment, dermatitis, headache, cognitive dysfunction, and muscle fibrosis.^2,3,4^ In addition, the heterogeneity among patients with advanced oral cancer complicates treatment planning, and the treatment decision is reached after discussion between patients and physicians.^5^ Risk stratification for patients with advanced cancer is crucial because it can be used to tailor the treatment to deintensify chemoradiotherapy for patients in the low-risk group or to intensify chemoradiotherapy for those in the high-risk group.^6,7,8^ Moreover, precise risk stratification is associated with improved allocation and use of health care resources. This information can be further used for care coordination and improving the use of health care resources.^9^

For precise treatment planning, tumor histologic information, such as TNM or staging, can be used for providing prognostic information.^10^ Moreover, the gene variant profile demonstrates the possibility of indicating cancer prognosis through statistical data mining and machine learning (ML) techniques.^11,12,13^ Recently, developing an ML-based model incorporating TNM data was associated with a promising clinical effect.^14^ Statistical data mining and ML are excellent analytical methods for classification through identification of data patterns from complex data.^15^ Statistical data mining and ML have demonstrated their successful applications in the medical field.^16,17,18,19^ The precise estimation of prognosis by using clinicopathologic and genetic information, including clinical data, pathologic data, and the gene variant profile, would provide a comprehensive disease overview.^10^ Given the trans-omic data, it is reasonable to harness the ML technologies, which are efficient at handling numerous predictors to generate a risk stratification model.

Here, we propose an elastic net penalized Cox proportional hazards regression–based risk stratification model to learn the patterns of different risk levels in cancer-specific survival, distant metastasis–free survival, and locoregional recurrence–free survival for postoperative patients with advanced oral cancer. According to the real-world database validation, our risk stratification models can be used as an online calculator by inputting the required data (eAppendix in the Supplement).

## Methods

### Data Source

We acquired data from a previously published study.^11^ In total, 345 patients with oral squamous cell carcinoma were retrospectively recruited from Chang Gung Memorial Hospital in Taoyuan, Taiwan, between January 1, 1996, and December 31, 2011. All patients had been followed up for 30 months or until death. No patients were lost to follow-up under the enrollment criteria. Details regarding inclusion and exclusion criteria are described in a previously published study.^11^ In brief, tumor samples were obtained from patients with stage III or IV node-positive cancer. The staging and pathologic diagnosis were assessed according to the criteria of the seventh edition of the American Joint Committee on Cancer.^20^ The patients had not been treated for oral squamous cell carcinoma before the tumor samples were obtained. No metastatic disease was documented when the tumor sample was obtained during surgery as well. Treatment choices (surgery alone, surgery with adjuvant radiotherapy, and surgery with adjuvant concurrent chemoradiotherapy [CCRT]) were determined for each patient according to the National Comprehensive Cancer Network (before 2008) or Chang Gung guidelines (2008).^11,21^ The study protocol was reviewed and approved by the Chang Gung Memorial Hospital Institutional Review Board, which waived patient consent because this was a retrospective study. We followed the Standards for Reporting of Diagnostic Accuracy (STARD) reporting guideline and the Transparent Reporting of a Multivariable Prediction Model for Individual Prognosis or Diagnosis (TRIPOD) reporting guideline.

Tumor samples were obtained during surgery for the following experiments of gene sequencing. The detailed settings of sample preparation and gene sequencing have been described in the previously published studies.^11,21^ In brief, ultra-deep sequencing of 44 cancer-related gene variant profiles was analyzed using the Ion 318 chip on the Ion Torrent PGM (Personal Genome Machine) system (Thermo Fisher Scientific), in which hg19 reference genome was used as the reference. The 44 cancer-related gene variant profiles were *ABL1* (OMIM 189980), *AKT1* (OMIM 164730), *ALK* (OMIM 105590), *APC* (OMIM 611731), *ATM* (OMIM 607585), *BRAF* (OMIM 164757), *CDH1* (OMIM 192090), *CDKN2A* (OMIM 600160), *CSF1R* (OMIM 164770), *CTNNB1* (OMIM 116806), *EGFR* (OMIM 131550), *ERBB2* (OMIM 164870), *ERBB4* (OMIM 600543), *FBXW7* (OMIM 606278), *FGFR1* (OMIM 136350), *FGFR2* (OMIM 176943), *FGFR3* (OMIM 134934), *FLT3* (OMIM 136351), *HNF1A* (OMIM 142410), *HRAS* (OMIM 190020), *IDH1* (OMIM 147700), *JAK3* (OMIM 600173), *KDR* (OMIM 191306), *KIT* (OMIM 164920), *KRAS* (OMIM 190070), *MET* (OMIM 164860), *MLH1* (OMIM 120436), *MPL* (OMIM 159530), *NOTCH1* (OMIM 190198), *NPM1* (OMIM 164040), *NRAS* (OMIM 164790), *PDGFRA* (OMIM 173490), *PIK3CA* (OMIM 171834), *PTEN* (OMIM 601728), *PTPN11* (OMIM 176876), *RB1* (OMIM 614041), *RET* (OMIM 164761), *SMAD4* (OMIM 600993), *SMARCB1* (OMIM 601607), *SMO* (OMIM 601500), *SRC* (OMIM 190090), *STK11* (OMIM 602216), *TP53* (OMIM 191170), and *VHL* (OMIM 608537). Sanger sequencing or pyrosequencing was used for confirming variants detected using the Torrent Variant Caller plug-in, version 3.2 (Thermo Fisher Scientific). The genetic features were obtained by next-generation sequencing using an ultra-deep (>1000×) sequencing approach for the primary tumor samples, examining more than 1200 nonsynonymous variants containing missense, nonsense, indel, and splicing types of the variant.

Information on the comprehensive clinical, pathologic, and genetic features of the patients was collected (Table). The comprehensive clinicopathologic and genetic features consisted of 5 clinical features (ie, sex, age at onset, alcohol drinking, betel quid chewing, and cigarette smoking), 17 pathologic features (eg, cancer primary site, pathologic T stage, pathologic N stage, pathologic stage, differentiation, pathologic tumor invasion depth, and nearest macroscopic margin), and 44 gene features (ie, 44 cancer-related genes).

**Table.** Characteristics of Patients With Oral Squamous Cell Carcinoma Who Underwent Surgery, Surgery With Adjuvant RT, or Surgery With Adjuvant CCRT

Characteristic	Treatment, No. (%)			*P* value
	Surgery alone (25 [7.5])	Surgery with adjuvant		
		RT (98 [29.3])	CCRT (211 [63.2])	
Sex				
Male	23 (92.0)	93 (94.9)	199 (94.3)	.86
Female	2 (8.0)	5 (5.1)	12 (5.7)	
Age at onset, median (IQR), y	50 (39-60)	48 (42-60)	48 (43-55)	.80
Alcohol drinking	16 (64.0)	64 (65.3)	162 (76.8)	.07
Betel quid chewing	17 (68.0)	81 (82.7)	175 (82.9)	.18
Cigarette smoking	23 (92.0)	89 (90.8)	192 (91.0)	.98
Cancer primary site				
Tongue	12 (48.0)	35 (35.7)	78 (37.0)	.06
Mouth floor	1 (4.0)	6 (6.1)	6 (2.8)	
Lip	0	1 (1.0)	1 (0.5)	
Buccal	8 (32.0)	41 (41.8)	79 (37.4)	
Gum (alveolar ridge)	3 (12.0)	10 (10.2)	31 (14.7)	
Hard palate	0	5 (5.1)	1 (0.5)	
Retromolar trigone	1 (4.0)	0	15 (7.1)	
Pathologic T stage				
1	3 (12.0)	4 (4.1)	8 (3.8)	.21
2	10 (40.0)	44 (44.9)	82 (38.9)	
3	7 (28.0)	20 (20.4)	38 (18.0)	
4	5 (20.0)	30 (30.6)	83 (39.3)	
Pathologic N stage				
1	14 (56.0)	62 (63.3)	44 (20.9)	<.001
2a	0	1 (1.0)	2 (0.9)	
2b	10 (40.0)	26 (26.5)	143 (67.8)	
2c	1 (4.0)	9 (9.2)	22 (10.4)	
Pathologic stage				
III	13 (52.0)	43 (43.9)	27 (12.8)	<.001
IV	12 (48.0)	55 (56.1)	184 (87.2)	
Differentiation				
Well differentiated	4 (16.0)	23 (23.5)	31 (14.7)	.09
Moderately differentiated	15 (60.0)	66 (67.3)	140 (66.4)	
Poorly differentiated	6 (24.0)	9 (9.2)	40 (19.0)	
Pathologic tumor invasion depth, median (IQR), mm	10 (5-15)	12 (9-18)	13 (8-19)	.17
Nearest microscopic margin, median (IQR), mm	7 (5-9)	8.5 (6-10)	8 (5-10)	.15
Total dissected lymph nodes, median (IQR), No.	35.0 (29.0-53.0)	38.5 (28.0-52.2)	47.0 (36.0-61.0)	<.001
Positive lymph nodes on dissection, median (IQR), No.	1 (1-3)	1 (1-3)	3 (2-4)	<.001
Lower neck lymph node (level IV or V) involvement	2 (8.0)	3 (3.1)	22 (10.4)	.09
Extranodal extension	10 (40.0)	25 (25.5)	161 (76.3)	<.001
Perineural invasion	5 (20.0)	46 (46.9)	122 (57.8)	.001
Lymphatic vessel invasion	0	13 (13.3)	30 (14.2)	.13
Vascular invasion	0	3 (3.1)	14 (6.6)	.20
Skin invasion	2 (8.0)	9 (9.2)	25 (11.8)	.70
Bone marrow invasion	2 (8.0)	22 (22.4)	44 (20.9)	.27
Genetic features				
* TP53*	16 (64.0)	62 (63.3)	140 (66.4)	.86
* PIK3CA*	6 (24.0)	20 (20.4)	44 (20.9)	.92
* CDKN2A*	2 (8.0)	11 (11.2)	29 (13.7)	.64
* HRAS*	8 (32.0)	6 (6.1)	16 (7.6)	<.001
* BRAF*	5 (20.0)	6 (6.1)	18 (8.5)	.09
* EGFR*	3 (12.0)	6 (6.1)	13 (6.2)	.53
* FGFR3*	3 (12.0)	6 (6.1)	10 (4.7)	.33
* SMAD4*	2 (8.0)	4 (4.1)	11 (5.2)	.72
* APC*	4 (16.0)	5 (5.1)	8 (3.8)	.03
* FGFR2*	3 (12.0)	3 (3.1)	8 (3.8)	.12
* MET*	1 (4.0)	4 (4.1)	8 (3.8)	.99
* KIT*	2 (8.0)	5 (5.1)	6 (2.8)	.34
* PTEN*	3 (12.0)	3 (3.1)	7 (3.3)	.09
* ERBB4*	2 (8.0)	3 (3.1)	8 (3.8)	.52
* RB1*	1 (4.0)	5 (5.1)	6 (2.8)	.61
* RET*	1 (4.0)	3 (3.1)	7 (3.3)	.97
* ATM*	1 (4.0)	4 (4.1)	6 (2.8)	.83
* NOTCH1*	1 (4.0)	2 (2.0)	7 (3.3)	.79
* ABL1*	3 (12.0)	4 (4.1)	4 (1.9)	.02
* SMO*	2 (8.0)	3 (3.1)	5 (2.4)	.30
* STK11*	1 (4.0)	2 (2.0)	7 (3.3)	.79
* FBXW7*	2 (8.0)	2 (2.0)	5 (2.4)	.23
* AKT1*	1 (4.0)	2 (2.0)	7 (3.3)	.79
* PDGFRA*	2 (8.0)	2 (2.0)	5 (2.4)	.23
* KDR*	1 (4.0)	1 (1.0)	6 (2.8)	.54
* CTNNB1*	1 (4.0)	1 (1.0)	5 (2.4)	.59
* PTPN11*	2 (8.0)	2 (2.0)	4 (1.9)	.16
* KRAS*	2 (8.0)	1 (1.0)	4 (1.9)	.09
* CDH1*	1 (4.0)	0	5 (2.4)	.24
* ERBB2*	0	0	4 (1.9)	.31
* SMARCB1*	0	0	6 (2.8)	.17
* JAK3*	1 (4.0)	0	3 (1.4)	.23
* FGFR1*	1 (4.0)	1 (1.0)	2 (0.9)	.41
* HNF1A*	0	2 (2.0)	1 (0.5)	.35
* MLH1*	1 (4.0)	0	3 (1.4)	.23
* VHL*	1 (4.0)	0	2 (0.9)	.17
* IDH1*	2 (8.0)	0	2 (0.9)	.004
* FLT3*	1 (4.0)	0	2 (0.9)	.17
* NRAS*	0	0	3 (1.4)	.41
* MPL*	1 (4.0)	0	1 (0.5)	.06
* NPM1*	1 (4.0)	1 (1.0)	0	.04
* ALK*	0	0	1 (0.5)	.75
* CSF1R*	0	0	1 (0.5)	.75
* SRC*	0	1 (1.0)	0	.30
Survival outcomes				
Cancer-specific survival	10 (40.0)	59 (60.2)	125 (59.2)	.18
Distant metastasis–free survival	17 (68.0)	76 (77.6)	154 (73.0)	.54
Locoregional recurrence–free survival	17 (68.0)	76 (77.6)	174 (82.5)	.16

Abbreviations: CCRT, concurrent chemoradiation; IQR, interquartile range; RT, radiotherapy.

### Model Development

Elastic net penalized Cox proportional hazards regression models were built using clinicopathologic and genetic features to identify the prognostic associations of the features and to calculate the survival index of each patient treated with different curative therapeutics^22,23,24^ (eFigure 1 in the Supplement). To examine whether prognostic associations of the features and the distribution of survival indices indicated different prognostic survival outcomes, we built a model for predicting 3 types of outcomes: cancer-specific survival, distant metastasis–free survival, and locoregional recurrence–free survival. A repeated, nested 3-fold cross-validation was applied to tune (inner cross-validation) and evaluate (outer cross-validation) the models (eFigure 1 in the Supplement). Regulation parameters (λ) and an elastic net mixing parameter (α) were selected by inner 3-fold cross-validation on the training set. In each outer fold, the median survival index in the training set was selected to divide patients in the test set into high-risk and low-risk groups. The models were developed using R software with the glmnet package (R Foundation for Statistical Computing).^22^ In addition, the performance of elastic net penalized Cox proportional hazards regression models were compared with the regular Cox proportional hazards regression model to evaluate the effects of elastic net penalty. We first built univariate Cox proportional hazards regression models for each clinicopathologic and genetic feature. The features associated with the outcomes (*P* < .05) were further used in the development of the multivariable Cox proportional hazards regression models. The median survival index was used to divide patients in the test set into high-risk and low-risk groups.

### Model Evaluation

For model evaluation, an outer 3-fold cross-evaluation was used to assess the performance of our models (eFigure 1 in the Supplement). The data were partitioned randomly into 3 sets, 1 set for testing and the other 2 sets for training. To evaluate the model stability, repeated nested cross-validation was performed 10 times for each outcome measurement. Thus, we generated 30 training and test sets to evaluate models for each type of prognostic survival and for each treatment method. Patients in the test set were classified into high-risk and low-risk groups based on their survival indices, with a threshold of median survival index in the training set. The log-rank test was used to compare the survival distributions between high-risk and low-risk groups. To evaluate the effectiveness of using comprehensive clinicopathologic and genetic features for model development, we compared the Akaike information criterion and the Harrell concordance index (C index)^25^ of models built using the clinicopathologic and genetic features with those using clinicopathologic features alone and genetic features alone.

### Feature Association Analysis

The associated prognostic clinicopathologic and genetic features were selected using elastic net penalized Cox proportional hazards regression models for 3 types of prognostic survival, and the coefficients were analyzed to evaluate the importance of the clinicopathologic and genetic features. On the basis of model development and evaluation approach (eFigure 1 in the Supplement), 30 models were built for each prognostic survival type. The number of times each feature was selected among the 30 models was used to evaluate the importance of the feature. The prognostic associations of the clinicopathologic and genetic features were defined as those of the features that were selected by more than 80% of the models (>24 of the 30 models). The hazard ratios of each feature, the exponential of the features’ coefficients, were used for comparing the association with the hazard rate of a given feature with a reference group.

### Statistical Analysis

Statistical analysis was conducted from February 1, 2018, to May 6, 2020. Analysis of variance was used for continuous data, and the Pearson χ^2^ test was used for categorical data. We performed repeated-measures analysis of variance with pairwise paired *t* test post hoc analyses and a nonparametric Friedman test with a pairwise paired Wilcoxon signed rank post hoc test on the Akaike information criterion and C index values of the models. The *P* values of pairwise comparison are adjusted using the Bonferroni multiple testing correction method. All statistical tests were 2-sided, and *P* < .05 was considered statistically significant. All analyses were performed using R software, version 3.4.0 (R Foundation for Statistical Computing).

## Results

### Patient Characteristics

Of 345 patients with oral squamous cell carcinoma who had clinical and next-generation sequencing data, 334 with complete data were included in the study. Of the 334 patients included in the analysis, the median age at onset was 48 years (interquartile range, 42-56 years), 315 patients (94.3%) were men, and the median follow-up duration was 55.0 months (interquartile range, 13-109 months). The Table shows the demographic, clinical, pathologic, and gene characteristics of the study population. In total, 211 patients (63.2%) underwent sugery with adjuvant CCRT, 98 (29.3%) underwent surgery with adjuvant radiotherapy, and 25 (7.5%) underwent surgery alone. Patients treated with postoperative adjuvant CCRT were likely to have the following risk factors: extranodal extension (161 of 211 [76.3%]; *P* < .001) and perineural invasion (122 of 211 [57.8%]; *P* = .001), high pathologic stages (stage IV, 184 of 211 [87.2%]; *P* < .001), and more total dissected lymph nodes (median, 47.0 [interquartile range, 36.0-61.0]; *P* < .001). The number of patients meeting cancer-specific survival outcomes was 194 (58.1%), the numbe of patients meeting distant metastasis–free survival outcomes was 247 (74.0%), and the number of patients meeting locoregional recurrence–free survival outcomes was 267 (79.9%).

### Performance of Risk Prediction Model

**Figure 1.**
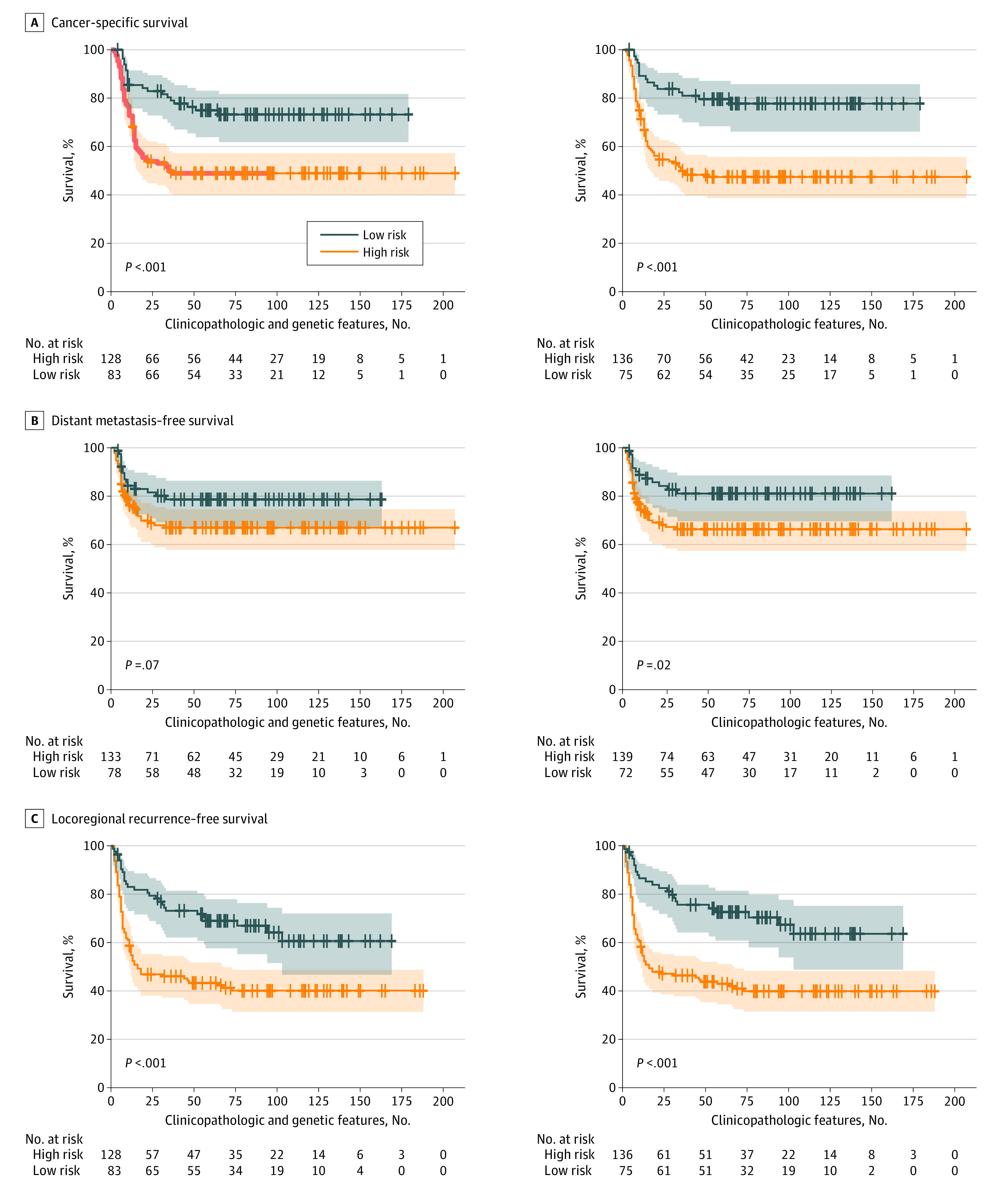
Kaplan-Meier Curves of Patients Who Received Postoperative Adjuvant Concurrent Chemoradiotherapy Stratified Using Elastic Net Penalized Cox Proportional Hazards Regression Models Built With Clinicopathologic and Genetic Features vs Clinicopathologic Features Alone A, Cancer-specific survival. B, Distant metastasis–free survival. C, Locoregional recurrence–free survival. The shaded areas indicate 95% CIs.

**Figure 2.**
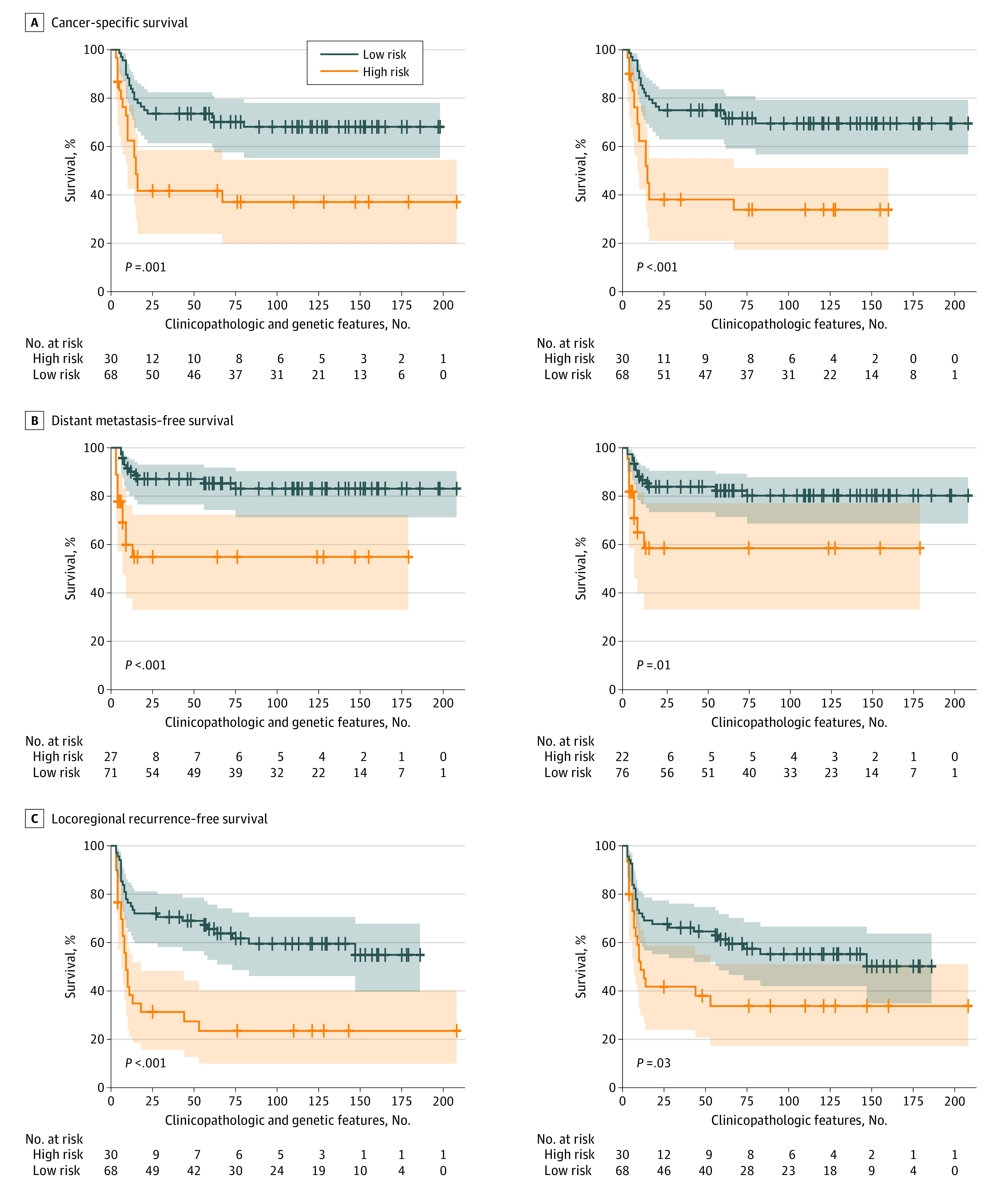
Kaplan-Meier Curves of Patients Who Received Postoperative Adjuvant Radiotherapy Stratified Using Elastic Net Penalized Cox Proportional Hazards Regression Models Built With Clinicopathologic and Genetic Features vs Clinicopathologic Alone A, Cancer-specific survival. B, Distant metastasis–free survival. C, Locoregional recurrence–free survival. The shaded areas indicate 95% CIs.

**Figure 3.**
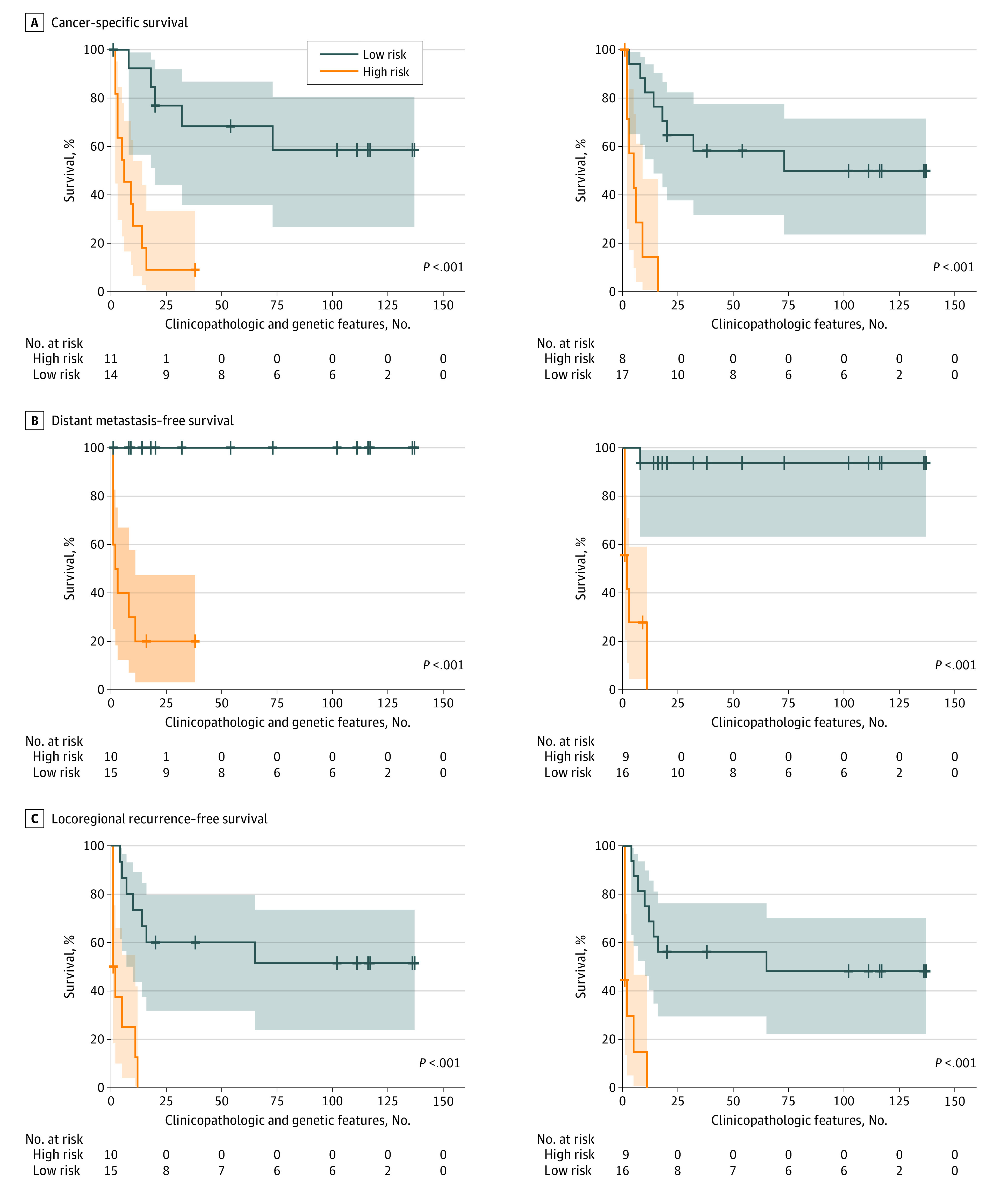
Kaplan-Meier Curves of Patients Who Underwent Surgery Alone Stratified Using Elastic Net Penalized Cox Proportional Hazards Regresssion Models Built With Clinicopathologic and Genetic Features vs Clinicopathologic Features Alone A, Cancer-specific survival. B, Distant metastasis–free survival. C, Locoregional recurrence–free survival. The shaded areas indicate 95% CIs.

The models built using clinicopathologic and genetic features successfully stratified patients who received postoperative CCRT (Figure 1; eFigure 2 in the Supplement [among 10 rounds of tests, only the first round of test results were plotted]), patients who received postoperative radiotherapy (Figure 2; eFigure 3 in the Supplement [the first round of test results]), and patients who received surgery alone (Figure 3; eFigure 4 in the Supplement [the first round of test results]) for cancer-specific survival and locoregional recurrence–free survival based on their survival indices. The number of patients and their follow-up durations in high-risk and low-risk groups for each survival outcome predicted by the models built using clinicopathologic and genetic features are shown in eTable 1 in the Supplement. The mean (SD) C indices of models for patients treated with postoperative adjuvant CCRT were 0.689 (0.050) for cancer-specific survival prediction, 0.702 (0.056) for distant metastasis–free survival prediction, and 0.693 (0.039) for locoregional recurrence–free survival prediction. For cancer-specific survival and locoregional recurrence–free survival prediction for patients treated with postoperative adjuvant CCRT, the C indices of the models built using clinicopathologic and genetic features were reported to be higher compared with those using clinicopathologic features alone (cancer-specific survival: mean [SD] C index, 0.689 [0.050] vs 0.673 [0.051]; *P* = .02; locoregional recurrence–free survival: mean [SD] C index, 0.693 [0.039] vs 0.678 [0.035]; *P* = .004); however, the classification performance in distant metastasis–free survival was not different (mean [SD] C index, 0.702 [0.056] vs 0.688 [0.048]; *P* = .09) (eTable 3 in the Supplement). Furthermore, these models built using clinicopathologic and genetic features fit better than the models built using genetic features in cancer-specific survival and locoregional recurrence–free survival (eTable 2 in the Supplement). The elastic net penalized Cox proportional hazards regression models outperformed regular Cox proportional hazards regression models in cancer-specific survival (C index, 0.689 vs 0.616; *P* < .001), distant metastasis–free survival (0.702 vs 0.614; *P* < .001), and locoregional recurrence–free survival (0.693 vs 0.650; *P* = .001).

### Features Associated With Prognostic Prediction

The prognostic associations of the clinicopathologic and genetic features were defined as those that were selected by more than 80% of the models (>24 of the 30 models). The essential features selected by the models for predicting prognostic survival types and their hazard ratios are shown in eFigure 5 and eTable 4 in the Supplement. Extranodal extension, positive lymph nodes on dissection, and *HRAS* variant were selected among the prognostic models for all types of prognostic survival measurements within the different treatment groups (eTable 4 in the Supplement).

### Risk Stratification in Patients With Postoperative Adjuvant CCRT

**Figure 4.**
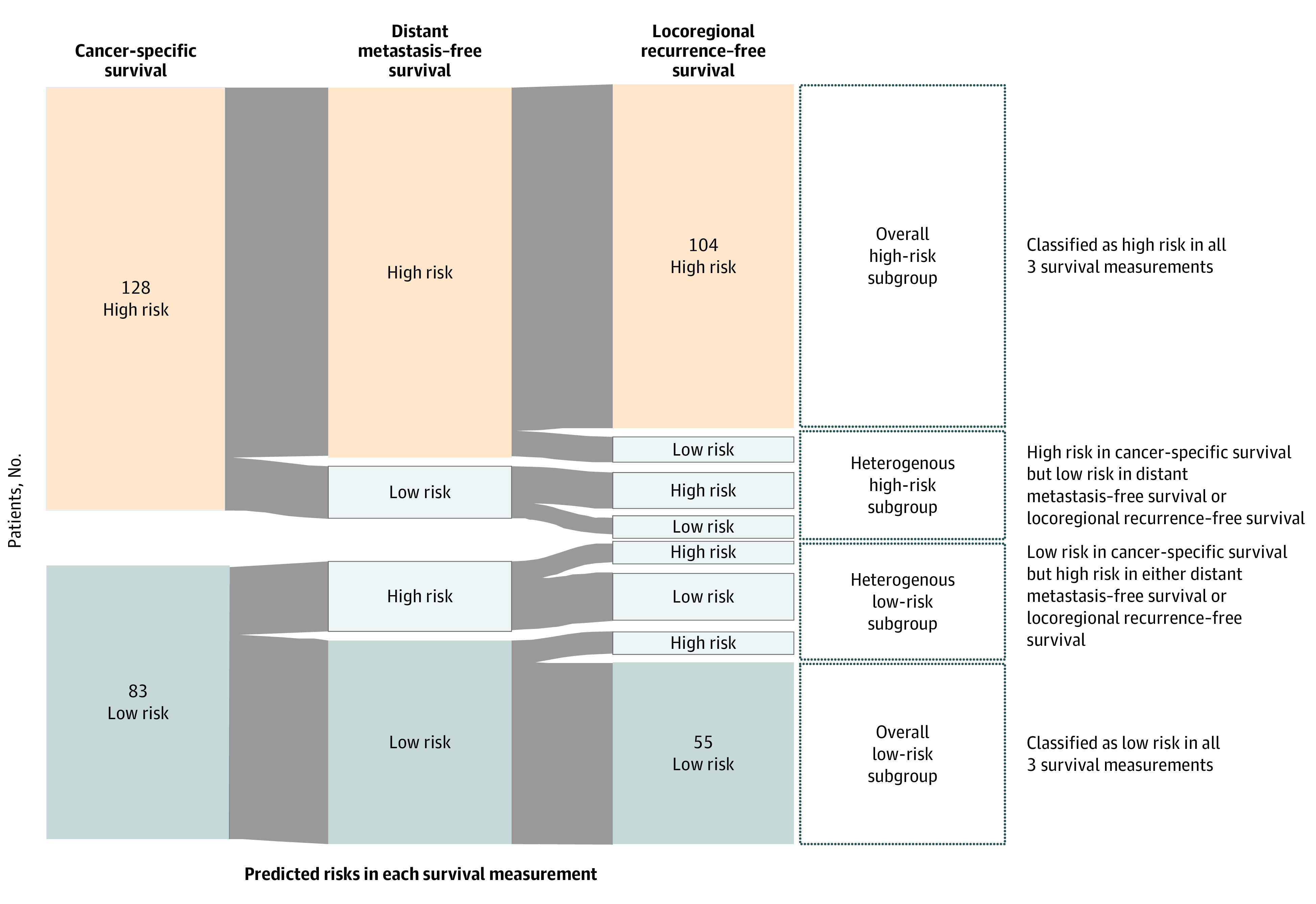
Risk Stratification From the Classification Result of Patients Who Underwent Surgery With Adjuvant Concurrent Chemoradiotherapy

A risk stratification result for patients who received postoperative adjuvant CCRT was visualized with the pattern of the predicted results from the 3 survival measurements (Figure 4). The risk predicted by the models using the cancer-specific survival measurement can represent the overall mortality for the patient. Then, the risk of the other measurements can further represent the risk of cancer metastasis and local recurrence. With risk classified by 3 survival measurements together, the patients can be further categorized into 4 subgroups—overall low-risk subgroup, heterogeneous low-risk subgroup, heterogeneous high-risk subgroup, and overall high-risk subgroup. The patients in the overall low-risk subgroup were classified as low risk in all survival measurements, and the patients in the overall high-risk subgroup were classified as high risk in all survival measurements. The patients in the heterogeneous low-risk subgroup were classified as low risk in cancer-specific measurement but with at least 1 high-risk classification in other measurements, which means that the patient had relative low risk in mortality but with different risks in the event of cancer metastasis or local recurrence. Among 211 patients with postoperative adjuvant CCRT, 104 patients were classified as being in the overall high-risk subgroup and 55 patients were classified as being in the overall low-risk subgroup.

## Discussion

In this study, we aligned the clinical scenario of cancer treatment end points with the ML technique to conduct risk stratification for patients with advanced oral cavity squamous cell carcinoma. The ML model provides information on personalized risk stratification for locoregional recurrence, distant metastasis, and cancer-specific survival. Clinical physicians can intensify or deintensify follow-up durations and treatments based on risk stratifications for postoperative patients with advanced oral cancer. Our risk stratification models were trained and validated based on an East Asian population, in which the prevalence of oral cavity cancer is much higher because of culture, behavior, and socioeconomic status.^26^ This work can fill the gap due to a lack of a prognosis prediction tool for the Asian population.

A prognostic prediction for patients with cancer could have high accuracy when comprehensive information is used in the predictive model. Several head and neck cancer prognosis calculators, such as the Maastro Clinic, LifeMath, Leiden,^27^ MyCancerJourney, Memorial Sloan Kettering, and Knight, have been published for the populations of the United States and the Netherlands.^28,29^ However, these risk calculators adopted clinical information only, without pathologic and genetic features. The gene variant profile containing variant measurements for multiple genes is another promising approach to estimate the behavior of a tumor. Using the powerful analytical capability of next-generation sequencing, a parallel analysis of multiple genes is possible. However, cancer development is a complex interaction between tumor cells, paratumor tissues, and other host factors.^1^ Thus, genetic features alone would not provide the whole picture of cancer, although gene features alone have proven useful in distinguishing between high-risk and low-risk individuals.^11^ In this work, we demonstrated that an improved level of precision of risk stratification is associated with the use of comprehensive clinicopathologic and genetic data (namely, clinical, pathologic, and genetic features) in terms of the Akaike information criterion (Figure 1, Figure 2, and Figure 3; eTable 2 in the Supplement). Moreover, according to the intrinsic feature selection of elastic net penalized Cox proportional hazards regression models, only 6 of 44 tumor-related gene variant profiles were selected in risk stratification models (eFigure 5 in the Supplement). In our models, the number of genes needed to be measured was largely reduced to 6, and multiplex polymerase chain reaction but not next-generation sequencing would be sufficient to detect gene variants. The cost of gene tests in our models would be considerably reduced, and the models would become affordable to patients with oral cancer who had suboptimal socioeconomic status.^26^ With the use of existing clinical and pathologic data, combined with the need fora fewer number of genes to be tested, the risk stratification models can be directly integrated into the current workflow of managing postoperative patients with advanced oral cancer.

Nested repeated cross-validation was used in our work, which is suitable for small data sets to provide an unbiased estimation of the performance of the prediction model and the importance of the feature.^30,31,32^ Machine learning can be a powerful tool if physicians participate in the model development process to align and fulfill the clinical purpose. A generalizable approach and locally relevant data, but not a generalized model, are warranted for a clinically applicable ML model.^16,33^

Combining the predicted risk classifications from the 3 survival measurements can provide a practical application of risk stratification (Figure 4). If a patient is classified as high risk in the predicted model based on a cancer-specific survival measurement, that may imply a more intensified need for outpatient and laboratory follow-up.^34^ Furthermore, the risk classification in the predicted models of locoregional recurrence–free survival can provide additional useful information on the tailored management of chemotherapy, and the risk classification in the predicted models of distant metastasis–free survival can provide additional useful information on the tailored management of radiotherapy. For patients with postoperative adjuvant CCRT, typically the follow-up plan would be identical without personalized risk stratification. The risk stratification demonstrated in Figure 4 provided a clinically relevant approach for personalized risk assessment.

### Limitations

Our work has some limitations. Our model was built and evaluated based on a relatively small, single, tertiary hospital–based retrospective cohort within an Asian population, which indicated a lack of generalizability to Western populations; model performance may differ when applied to data from other institutions. That is, the models might not be recommended for direct use in other institutions because of the high level of diversity of various factors. However, a nested, repeated, 3-fold cross-validation approach was used to minimize bias and imitate external validation. The workflow is generic and can be applied to different institutions. A prospective, multicenter trial is required to validate the utility of the risk stratification model in future studies. Although the predictive model had a relatively stable performance, some contradictory results were observed wherein the patient was simultaneously classified into high-risk and low-risk groups in different prognostic survival outcomes. In addition, the surgical procedure for advanced head and neck cancer was highly complicated and often involved reconstructive surgery, which may affect the patient’s outcome.^19^

## Conclusions

In this prognostic cohort study, we developed and validated risk stratification models for postoperative patients with advanced oral cancer by using comprehensive clinicopathologic and genetic data. The risk stratification models that aligned clinical treatment scenarios with the ML technique may indicate the prognostic risks of locoregional recurrence, distant metastasis, and cancer-specific survival. Accurate risk stratification by use of ML models with an online calculator may facilitate a more precise management of cases of advanced oral cancer.

## Notes

Supplement. **eFigure 1.** Elastic-Net Penalized Cox Proportional Hazards Regression Model Development and Evaluation Flowchart **eFigure 2.** Kaplan-Meier Curves of Patients Who Underwent Postoperative Adjuvant Concurrent Chemoradiotherapy Stratified Using Elastic Net Penalized Cox Proportional Hazards Regression Models Built With Genetic Features Alone **eFigure 3.** Kaplan-Meier Curves of Patients Who Underwent Postoperative Adjuvant Radiotherapy Stratified Using Elastic Net Penalized Cox Proportional Hazards Regression Models Built With Genetic Features Alone **eFigure 4.** Kaplan-Meier Curves of Patients Who Underwent Surgery Alone Stratified Using Elastic Net Penalized Cox Proportional Hazards Regression Models Built With Genetic Features Alone **eFigure 5.** Features Associated With Prognostic Prediction **eTable 1.** Follow-up Duration in Each Group of Patients **eTable 2.** Akaike Information Criterion Comparison of the Elastic Net Penalized Cox Proportional Hazards Regression Models Built With Comprehensive Clinicopathologic and Genetic Features vs Clinicopathologic Features Alone and Genetic Features **eTable 3.** Harrell’s Concordance Index Comparison of the Elastic Net Penalized Cox Proportional Hazards Regression Models Built With Comprehensive Clinicopathologic and Genetic Features vs Clinicopathologic Features Alone and Genetic Features **eTable 4.** Features Associated With Prognostic Prediction and the Hazard Ratios **eAppendix.** Online Calculator for Survival Risk Stratification in Patients With Advanced Oral Cancer

## References

[1] Chinn SB, Myers JN Oral cavity carcinoma: current management, controversies, and future directions. J Clin Oncol. 2015;33(29):3269-3276. doi:10.1200/JCO.2015.61.2929 26351335 PMC5320919

[2] Salama JK, Stenson KM, List MA, Characteristics associated with swallowing changes after concurrent chemotherapy and radiotherapy in patients with head and neck cancer. Arch Otolaryngol Head Neck Surg. 2008;134(10):1060-1065. doi:10.1001/archotol.134.10.1060 18936351

[3] Langerman A, Maccracken E, Kasza K, Haraf DJ, Vokes EE, Stenson KM Aspiration in chemoradiated patients with head and neck cancer. Arch Otolaryngol Head Neck Surg. 2007;133(12):1289-1295. doi:10.1001/archotol.133.12.1289 18086974

[4] Brailo V, Boras VV, Juras DV, Rogulj AA, Brzak BL, Alajbeg I Oral side effects of head and neck irradiation In: Akarslan Z, ed. Diagnosis and Management of Head and Neck Cancer. InTech; 2017. doi:10.5772/intechopen.68961

[5] Lo Nigro C, Denaro N, Merlotti A, Merlano M Head and neck cancer: improving outcomes with a multidisciplinary approach. Cancer Manag Res. 2017;9:363-371. doi:10.2147/CMAR.S115761 28860859 PMC5571817

[6] Pearlstein KA, Wang K, Amdur RJ, Quality of life for patients with favorable-risk HPV-associated oropharyngeal cancer after de-intensified chemoradiotherapy. Int J Radiat Oncol Biol Phys. 2019;103(3):646-653. doi:10.1016/j.ijrobp.2018.10.033 30395903

[7] Brana I, Siu LL Locally advanced head and neck squamous cell cancer: treatment choice based on risk factors and optimizing drug prescription. Ann Oncol. 2012;23(suppl 10):x178-x185. doi:10.1093/annonc/mds322 22987958

[8] Schuller DE, Ozer E, Agrawal A, Grecula JC, Rhoades CA, Young DC Multimodal intensification regimens for advanced, resectable, previously untreated squamous cell cancer of the oral cavity, oropharynx, or hypopharynx: a 12-year experience. Arch Otolaryngol Head Neck Surg. 2007;133(4):320-326. doi:10.1001/archotol.133.4.320 17438244

[9] Bates DW, Saria S, Ohno-Machado L, Shah A, Escobar G Big data in health care: using analytics to identify and manage high-risk and high-cost patients. Health Aff (Millwood). 2014;33(7):1123-1131. doi:10.1377/hlthaff.2014.0041 25006137

[10] Karadaghy OA, Shew M, New J, Bur AM Development and assessment of a machine learning model to help predict survival among patients with oral squamous cell carcinoma. JAMA Otolaryngol Head Neck Surg. 2019;145(12):1115-1120. doi:10.1001/jamaoto.2019.0981 31045212 PMC6499120

[11] Wang H-M, Liao C-T, Yen T-C, Clues toward precision medicine in oral squamous cell carcinoma: utility of next-generation sequencing for the prognostic stratification of high-risk patients harboring neck lymph node extracapsular extension. Oncotarget. 2016;7(39):63082-63092. doi:10.18632/oncotarget.11762 27590518 PMC5325348

[12] Chaudhary K, Poirion OB, Lu L, Garmire LX Deep learning–based multi-omics integration robustly predicts survival in liver cancer. Clin Cancer Res. 2018;24(6):1248-1259. doi:10.1158/1078-0432.CCR-17-0853 28982688 PMC6050171

[13] Schmidt S, Linge A, Zwanenburg A, ; DKTK-ROG Development and validation of a gene signature for patients with head and neck carcinomas treated by postoperative radio(chemo)therapy. Clin Cancer Res. 2018;24(6):1364-1374. doi:10.1158/1078-0432.CCR-17-2345 29298797

[14] Suchting R, Hébert ET, Ma P, Kendzor DE, Businelle MS Using elastic net penalized Cox proportional hazards regression to identify predictors of imminent smoking lapse. Nicotine Tob Res. 2019;21(2):173-179. doi:10.1093/ntr/ntx201 29059349 PMC7962780

[15] Azuaje F Witten IH, Frank E: *Data Mining: Practical Machine Learning Tools and Techniques* 2nd edition. Biomed Eng OnLine. 2006;5(1):51. doi:10.1186/1475-925X-5-51

[16] Tseng Y-J, Huang C-E, Wen C-N, Predicting breast cancer metastasis by using serum biomarkers and clinicopathological data with machine learning technologies. Int J Med Inform. 2019;128:79-86. doi:10.1016/j.ijmedinf.2019.05.003 31103449

[17] Wang H-Y, Hsieh C-H, Wen C-N, Wen Y-H, Chen C-H, Lu J-J Cancers screening in an asymptomatic population by using multiple tumour markers. PLoS One. 2016;11(6):e0158285. doi:10.1371/journal.pone.0158285 27355357 PMC4927114

[18] Lin W-Y, Chen C-H, Tseng Y-J, Predicting post-stroke activities of daily living through a machine learning–based approach on initiating rehabilitation. Int J Med Inform. 2018;111:159-164. doi:10.1016/j.ijmedinf.2018.01.002 29425627

[19] Rajkomar A, Dean J, Kohane I Machine learning in medicine. N Engl J Med. 2019;380(14):1347-1358. doi:10.1056/NEJMra1814259 30943338

[20] Edge SB, Compton CC The American Joint Committee on Cancer: the 7th edition of the AJCC cancer staging manual and the future of TNM. Ann Surg Oncol. 2010;17(6):1471-1474. doi:10.1245/s10434-010-0985-4 20180029

[21] Chen S-J, Liu H, Liao C-T, Ultra-deep targeted sequencing of advanced oral squamous cell carcinoma identifies a mutation-based prognostic gene signature. Oncotarget. 2015;6(20):18066-18080. doi:10.18632/oncotarget.3768 25980437 PMC4621868

[22] Friedman J, Hastie T, Tibshirani R Regularization paths for generalized linear models via coordinate descent. J Stat Softw. 2010;33(1):1-22. doi:10.18637/jss.v033.i01 20808728 PMC2929880

[23] Simon N, Friedman J, Hastie T, Tibshirani R Regularization paths for Cox’s proportional hazards model via coordinate descent. J Stat Softw. 2011;39(5):1-13. doi:10.18637/jss.v039.i05 27065756 PMC4824408

[24] Tibshirani R, Bien J, Friedman J, Strong rules for discarding predictors in lasso-type problems. J R Stat Soc Series B Stat Methodol. 2012;74(2):245-266. doi:10.1111/j.1467-9868.2011.01004.x 25506256 PMC4262615

[25] Harrell FE Jr, Lee KL, Mark DB Multivariable prognostic models: issues in developing models, evaluating assumptions and adequacy, and measuring and reducing errors. Stat Med. 1996;15(4):361-387. doi:10.1002/(SICI)1097-0258(19960229)15:4<361::AID-SIM168>3.0.CO;2-4 8668867

[26] Bray F, Ferlay J, Soerjomataram I, Siegel RL, Torre LA, Jemal A Global cancer statistics 2018: GLOBOCAN estimates of incidence and mortality worldwide for 36 cancers in 185 countries. CA Cancer J Clin. 2018;68(6):394-424. doi:10.3322/caac.21492 30207593

[27] Datema FR, Ferrier MB, Vergouwe Y, Update and external validation of a head and neck cancer prognostic model. Head Neck. 2013;35(9):1232-1237. doi:10.1002/hed.23117 22847987

[28] Prince V, Bellile EL, Sun Y, Individualized risk prediction of outcomes for oral cavity cancer patients. Oral Oncol. 2016;63:66-73. doi:10.1016/j.oraloncology.2016.11.005 27939002 PMC5193389

[29] Hoban CW, Beesley LJ, Bellile EL, Individualized outcome prognostication for patients with laryngeal cancer. Cancer. 2018;124(4):706-716. doi:10.1002/cncr.31087 29112231 PMC5800991

[30] Varma S, Simon R Bias in error estimation when using cross-validation for model selection. BMC Bioinformatics. 2006;7:91. doi:10.1186/1471-2105-7-91 16504092 PMC1397873

[31] Filzmoser P, Liebmann B, Varmuza K Repeated double cross validation. J Chemometr. 2009;23(4):160-171. doi:10.1002/cem.1225

[32] Krstajic D, Buturovic LJ, Leahy DE, Thomas S Cross-validation pitfalls when selecting and assessing regression and classification models. J Cheminform. 2014;6(1):10. doi:10.1186/1758-2946-6-10 24678909 PMC3994246

[33] Oh J, Makar M, Fusco C, A generalizable, data-driven approach to predict daily risk of *Clostridium difficile* infection at two large academic health centers. Infect Control Hosp Epidemiol. 2018;39(4):425-433. doi:10.1017/ice.2018.16 29576042 PMC6421072

[34] Mariotto AB, Noone A-M, Howlader N, Cancer survival: an overview of measures, uses, and interpretation. J Natl Cancer Inst Monogr. 2014;2014(49):145-186. doi:10.1093/jncimonographs/lgu024 25417231 PMC4829054

