# Supplementary material for: Development of a Machine Learning Model for Survival Risk Stratification of Patients With Advanced Oral Cancer
Source: JAMA Netw Open. 2020 Aug 21;3(8):e2011768. doi: 10.1001/jamanetworkopen.2020.11768 (PMC7442932; doi:10.1001/jamanetworkopen.2020.11768)
Supplement: Supplement. — eFigure 1. Elastic-Net Penalized Cox Proportional Hazards Regression Model Development and Evaluation Flowchart eFigure 2. Kaplan-Meier Curves of Patients Who Underwent Postoperative Adjuvant Concurrent Chemoradiotherapy Stratified Using Elastic Net Penalized Cox Proportional Hazards Regression Models Built With Genetic Features Alone eFigure 3. Kaplan-Meier Curves of Patients Who Underwent Postoperative Adjuvant Radiotherapy Stratified Using Elastic Net Penalized Cox Proportional Hazards Regression Models Built With Genetic Features Alone eFigure 4. Kaplan-Meier Curves of Patients Who Underwent Surgery Alone Stratified Using Elastic Net Penalized Cox Proportional Hazards Regression Models Built With Genetic Features Alone eFigure 5. Features Associated With Prognostic Prediction eTable 1. Follow-up Duration in Each Group of Patients eTable 2. Akaike Information Criterion Comparison of the Elastic Net Penalized Cox Proportional Hazards Regression Models Built With Comprehensive Clinicopathologic and Genetic Features vs Clinicopathologic Features Alone and Genetic Features eTable 3. Harrell’s Concordance Index Comparison of the Elastic Net Penalized Cox Proportional Hazards Regression Models Built With Comprehensive Clinicopathologic and Genetic Features vs Clinicopathologic Features Alone and Genetic Features eTable 4. Features Associated With Prognostic Prediction and the Hazard Ratios eAppendix. Online Calculator for Survival Risk Stratification in Patients With Advanced Oral Cancer [file jamanetwopen-3-e2011768-s001.pdf]

Supplementary Online Content

Tseng Y-J, Wang H-Y, Lin T-W, Lu J-J, Hsieh C-H, Liao C-T. Development of a machine learning model for survival risk stratification of patients with advanced oral cancer. *JAMA Netw Open.* 2020;3(8):e2011768. doi:10.1001/jamanetworkopen.2020.11768

eFigure 1. Elastic-Net Penalized Cox Proportional Hazards Regression Model Development and Evaluation Flowchart
eFigure 2. Kaplan-Meier Curves of Patients Who Underwent Postoperative Adjuvant Concurrent Chemoradiotherapy Stratified Using Elastic Net Penalized Cox Proportional Hazards Regression Models Built With Genetic Features Alone
eFigure 3. Kaplan-Meier Curves of Patients Who Underwent Postoperative Adjuvant Radiotherapy Stratified Using Elastic Net Penalized Cox Proportional Hazards Regression Models Built With Genetic Features Alone
eFigure 4. Kaplan-Meier Curves of Patients Who Underwent Surgery Alone Stratified Using Elastic Net Penalized Cox Proportional Hazards Regression Models Built With Genetic Features Alone
eFigure 5. Features Associated With Prognostic Prediction
eTable 1. Follow-up Duration in Each Group of Patients
eTable 2. Akaike Information Criterion Comparison of the Elastic Net Penalized Cox Proportional Hazards Regression Models Built With Comprehensive Clinicopathologic and Genetic Features vs Clinicopathologic Features Alone and Genetic Features
eTable 3. Harrell's Concordance Index Comparison of the Elastic Net Penalized Cox Proportional Hazards Regression Models Built With Comprehensive Clinicopathologic and Genetic Features vs Clinicopathologic Features Alone and Genetic Features
eTable 4. Features Associated With Prognostic Prediction and the Hazard Ratios
eAppendix. Online Calculator for Survival Risk Stratification in Patients With Advanced Oral Cancer

This supplementary material has been provided by the authors to give readers additional information about their work.

© 2020 Tseng Y-J et al. *JAMA Network Open.*

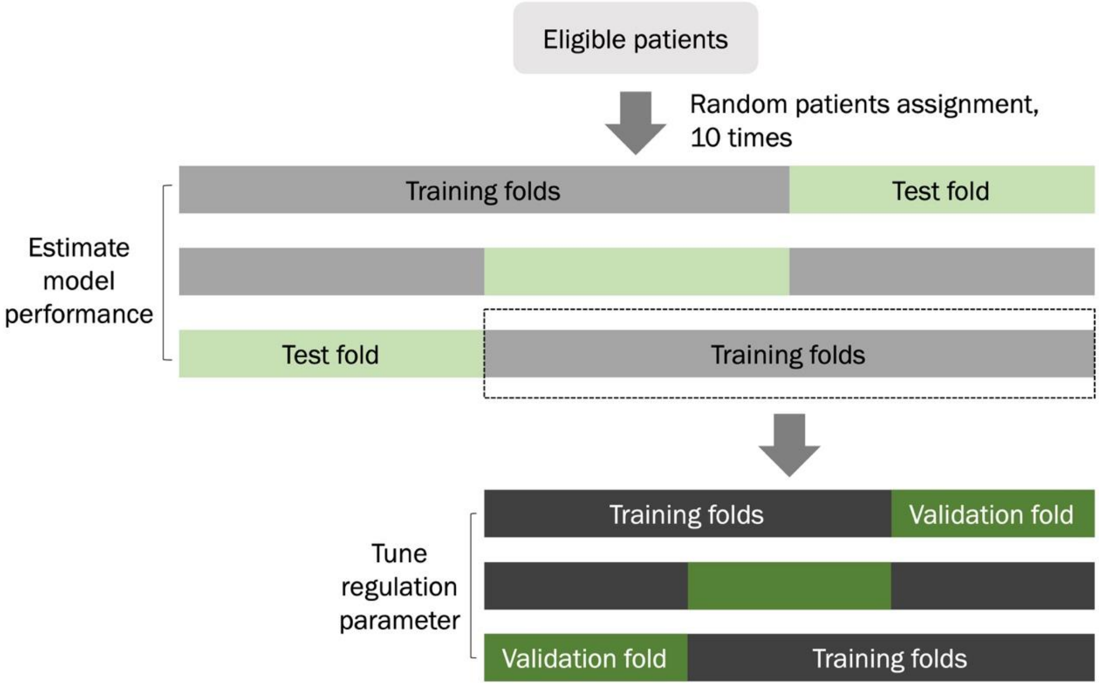

eFigure 1. Elastic-Net Penalized Cox Proportional Hazards Regression Model Development and Evaluation Flowchart

© 2020 Tseng Y-J et al. *JAMA Network Open.*

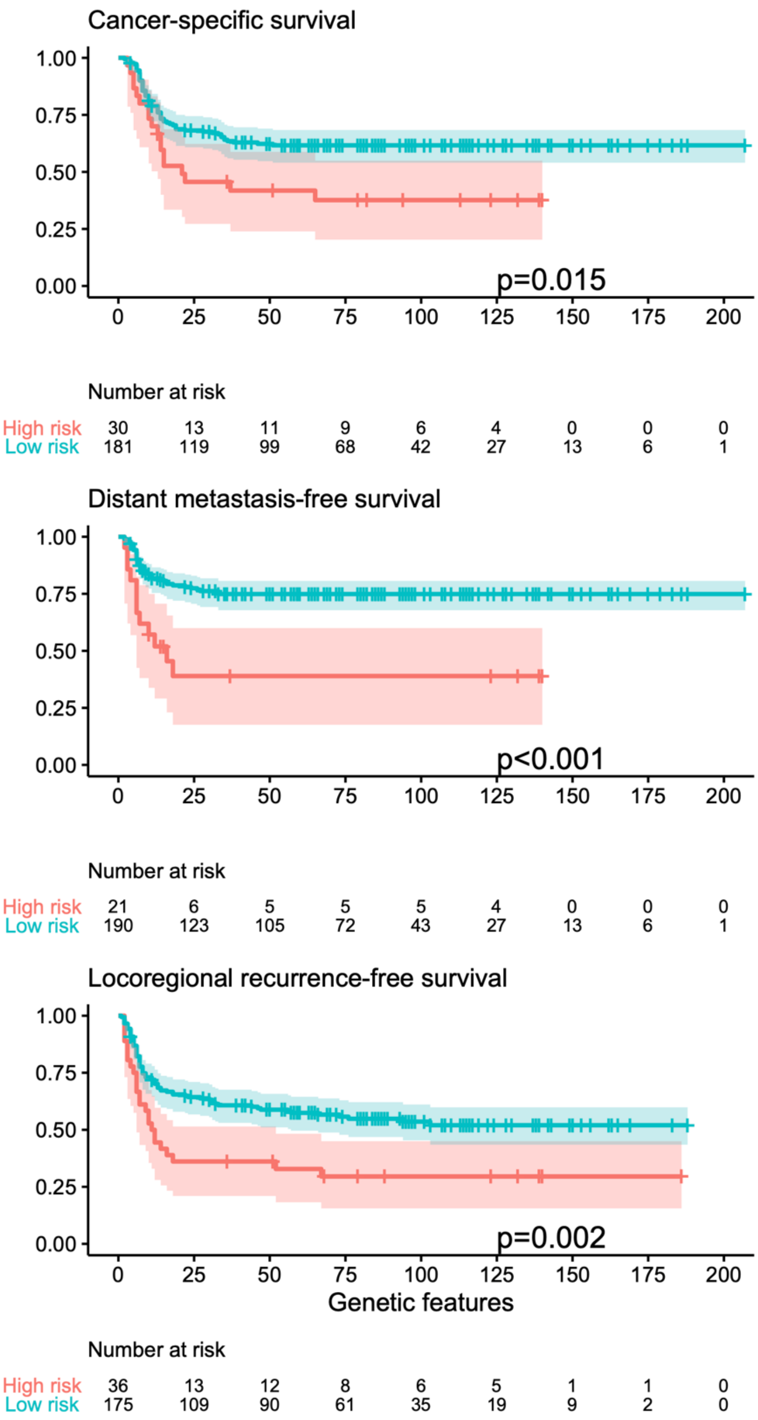

eFigure 2. Kaplan-Meier Curves of Patients Who Underwent Postoperative Adjuvant Concurrent Chemoradiotherapy Stratified Using Elastic Net Penalized Cox Proportional Hazards Regression Models Built With Genetic Features Alone

The models were built to predict the three types of prognostic survival outcomes, namely cancer-specific survival, distant metastasis-free survival, and locoregional recurrence-free survival.

© 2020 Tseng Y-J et al. *JAMA Network Open.*

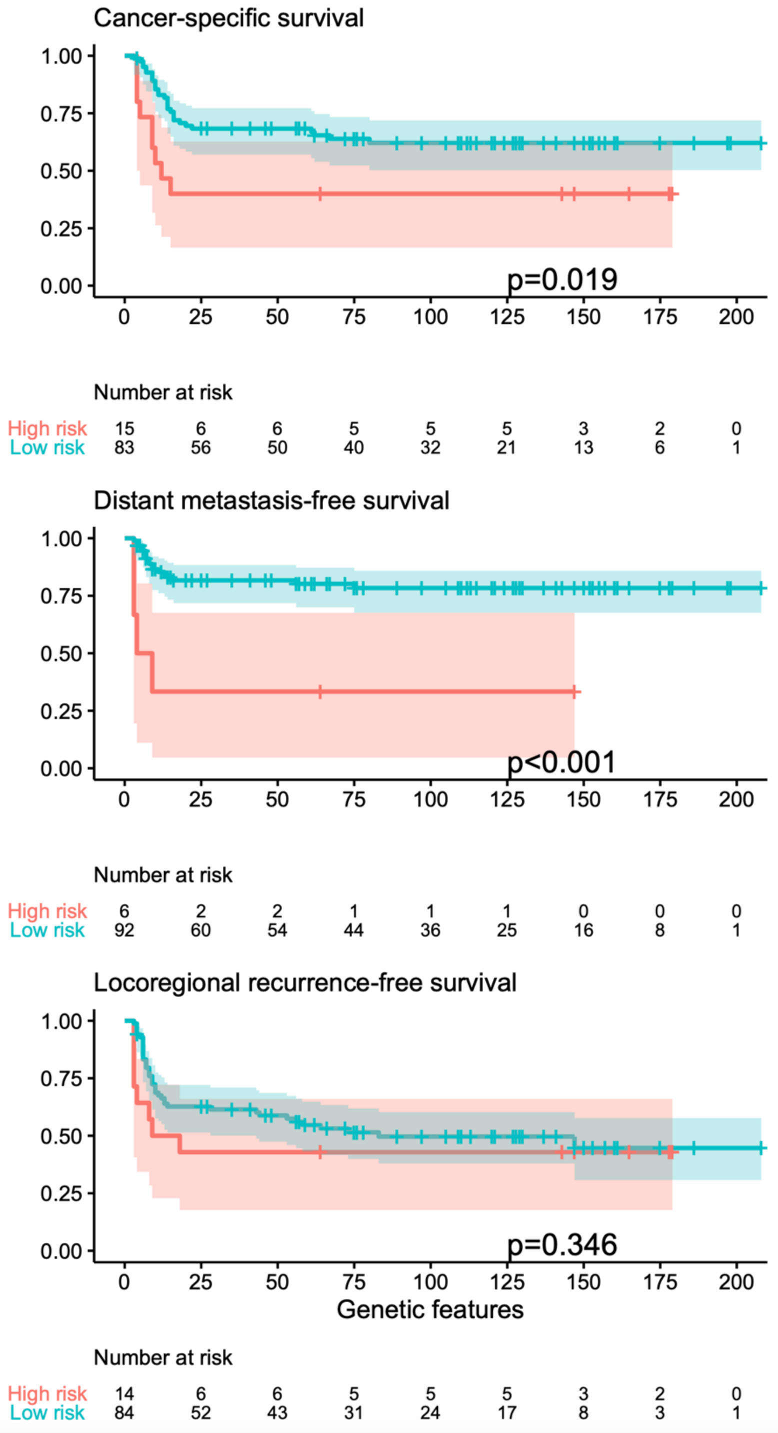

eFigure 3. Kaplan-Meier Curves of Patients Who Underwent Postoperative Adjuvant Radiotherapy Stratified Using Elastic Net Penalized Cox Proportional Hazards Regression Models Built With Genetic Features Alone

The models were built to predict the three types of prognostic survival outcomes, namely cancer-specific survival, distant metastasis-free survival, and locoregional recurrence-free survival.

© 2020 Tseng Y-J et al. *JAMA Network Open.*

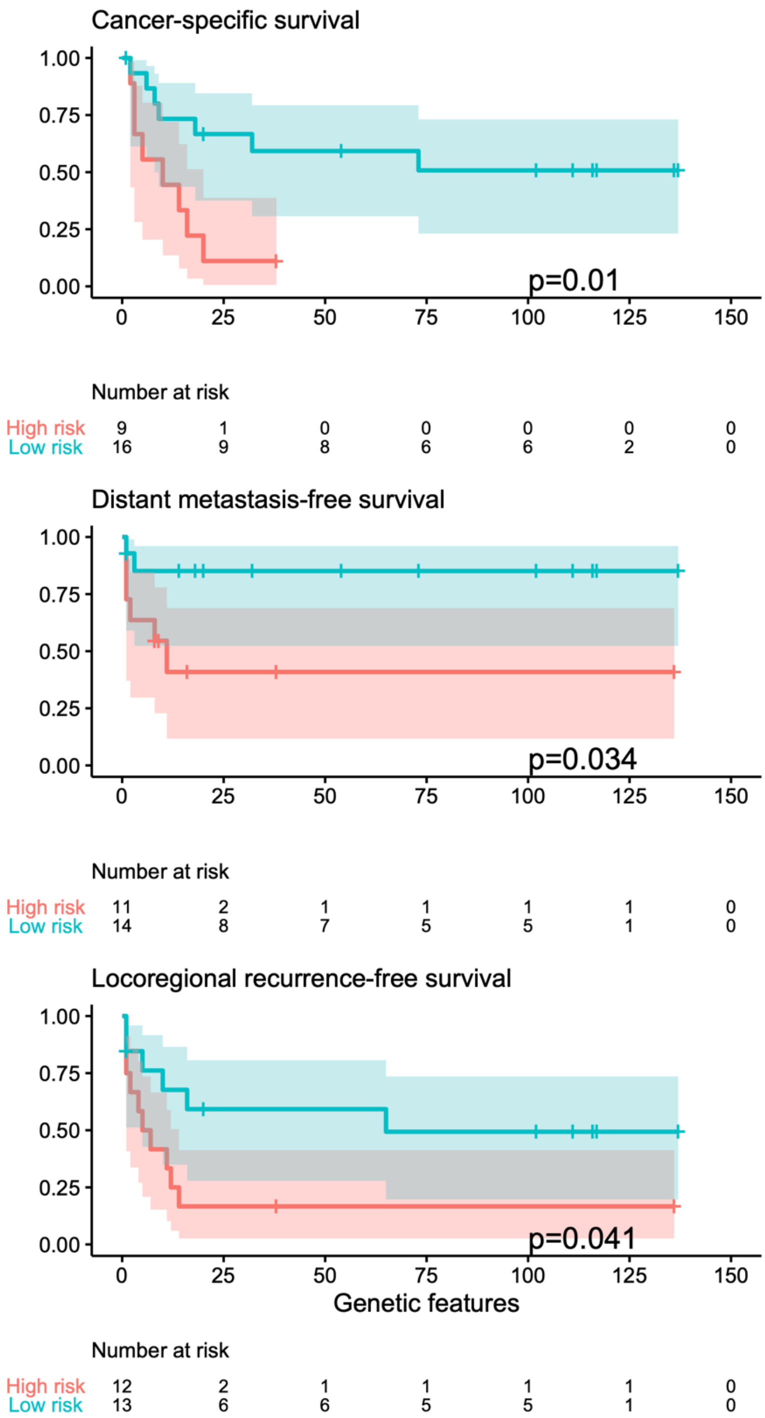

eFigure 4. Kaplan-Meier Curves of Patients Who Underwent Surgery Alone Stratified Using Elastic Net Penalized Cox Proportional Hazards Regression Models Built With Genetic Features Alone

The models were built to predict the three types of prognostic survival outcomes, namely cancer-specific survival, distant metastasis-free survival, and locoregional recurrence-free survival.

© 2020 Tseng Y-J et al. *JAMA Network Open.*

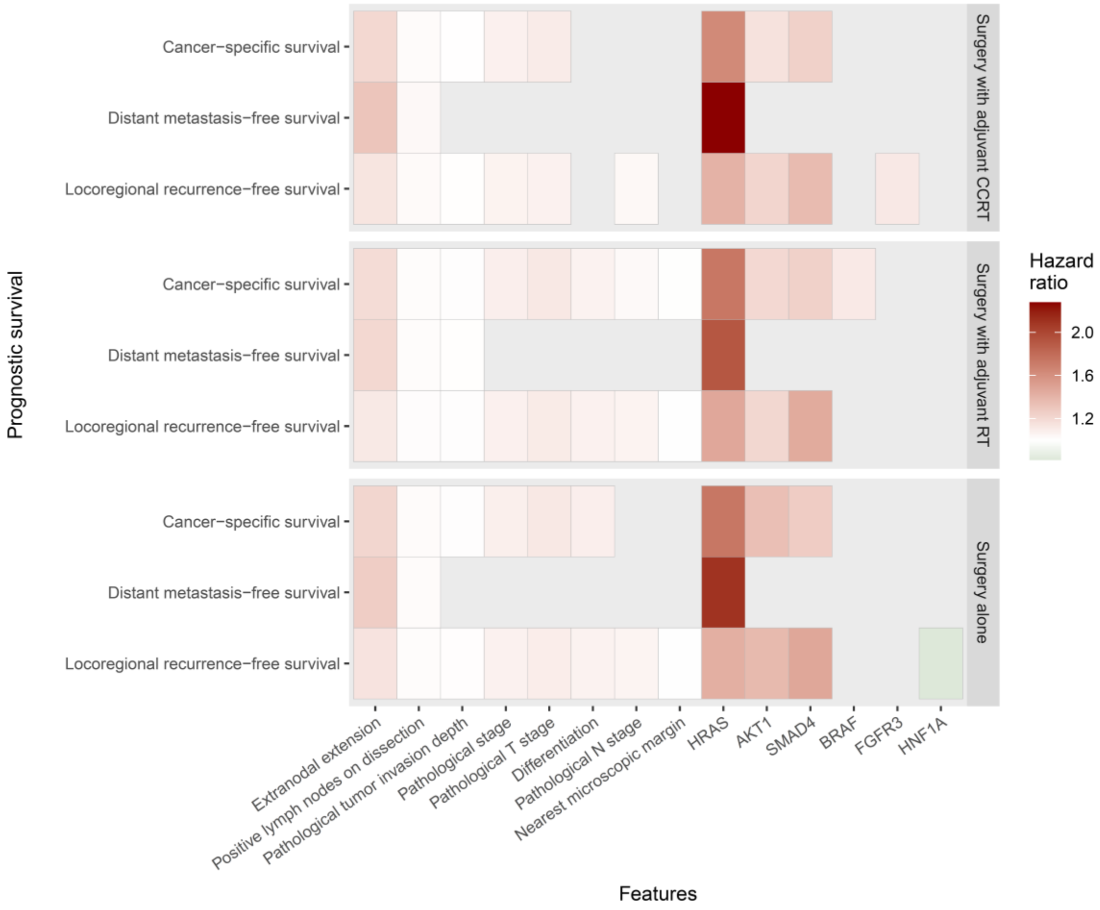

eFigure 5. Features Associated With Prognostic Prediction

The clinicopathologic and genetic features selected by more than 80% of the elastic-net penalized Cox's proportional hazards regression models are plotted in the figure. The block colors indicate the hazard ratios of clinicopathologic and genetic features in prognostic models. The feature is not selected in the specific model colored in gray. RT: radiotherapy; CCRT: concurrent chemoradiation.

© 2020 Tseng Y-J et al. *JAMA Network Open.*

eTable 1. Follow-up Duration in Each Group of Patients

The number of patients and their follow-up durations in high- and low-risk groups for each survival outcome divided by the models that were built using clinicopathologic and genetic features.

	Survival outcome for risk classification	Risk level	Case number	Follow-up duration, median (IQR)
Surgery with adjuvant CCRT[a]	Cancer-specific survival	High	128	32.5 (83.25)
		Low	83	65 (64.5)
	Distant metastasis-free survival	High	133	36 (85)
		Low	78	64.5 (74.75)
	Locoregional recurrence-free survival	High	128	15 (73.25)
		Low	83	64 (65)
Surgery with adjuvant RT[b]	Cancer-specific survival	High	30	15 (66.25)
		Low	68	84.5 (116.5)
	Distant metastasis-free survival	High	27	9 (40)
		Low	71	78 (108)
	Locoregional recurrence-free survival	High	30	9 (34.75)
		Low	68	65 (116.5)
Surgery alone	Cancer-specific survival	High	11	6 (9)
		Low	14	63.5 (94.75)
		High	10	2.5 (9.25)

© 2020 Tseng Y-J et al. *JAMA Network Open.*

	Distant metastasis-free survival	Low	15	54 (97.5)
	Locoregional recurrence-free survival	High	10	1 (3.25)
		Low	15	38 (101.5)

© 2020 Tseng Y-J et al. *JAMA Network Open.*

eTable 2. Akaike Information Criterion Comparison of the Elastic Net Penalized Cox Proportional Hazards Regression Models Built With Comprehensive Clinicopathologic and Genetic Features vs Clinicopathologic Features Alone and Genetic Features

Outcome	Akaike information criterion [mean (SD) or median (IQR)]			
	Using clinicopathologic and genetic features	Using clinicopathologic features	Using genetic features	P values<br>All features test<br>Paired test
Surgery with adjuvant CCRT				
Cancer-specific survival	851.634 (6.621)	856.638 (7.350)	870.004 (1.562)	<0.001 [a]<br>0.363 [c]
Distant metastasis-free survival	577.373 (4.513)	579.848 (1.651)	579.709 (2.403)	0.12 [a]<br>0.17 [c]
Locoregional recurrence-free survival	1009.879 (5.238)	1012.244 (4.246)	1026.193 (1.322)	<0.001 [a]<br>0.402 [c]
Surgery with adjuvant RT [e]				
Cancer-specific survival	331.160 (2.305)	331.160 (3.075)	337.088 (0.786)	<0.001 [b]<br>1 [d]
Distant metastasis-free survival	183.336 (2.879)	188.243 (2.686)	186.315 (2.659)	<0.001 [b]<br>0.006 [d]
Locoregional recurrence-free survival	407.532 (5.127)	405.148 (5.381)	414.525 (0.286)	<0.001 [b]<br>1 [d]
Surgery alone [e]				
Cancer-specific survival	71.634 (6.536)	65.703 (4.835)	76.491 (2.169)	<0.001 [b]<br>0.378 [d]

© 2020 Tseng Y-J et al. *JAMA Network Open.*

Distant metastasis-free survival	31.727 (1.448)	35.246 (4.925)	41.701 (1.894)	<0.001 [b] 0.017 [d]
Locoregional recurrence-free survival	76.622 (0)	75.285 (0)	86.389 (0.828)	<0.001 [b] 0.021 [d]

Abbreviations: CCRT, concurrent chemoradiation; RT, radiotherapy; SD, standard deviation

[a] Repeated ANOVA test, comparing three feature sets; [b] Friedman's test, comparing three feature sets

[c] Pairwise paired t-tests, comparing models using clinicopathologic and genetic features versus those that used clinicopathologic features, alone. P-values are adjusted using the Bonferroni multiple testing correction method.

[d] Pairwise paired Wilcoxon signed-rank test, comparing models using clinicopathologic and genetic features versus clinicopathologic features alone. P-values are adjusted using the Bonferroni multiple testing correction method.

[d] This data point is reported as the median (IQR)

© 2020 Tseng Y-J et al. *JAMA Network Open.*

eTable 3. Harrell's Concordance Index Comparison of the Elastic Net Penalized Cox Proportional Hazards Regression Models Built With Comprehensive Clinicopathologic and Genetic Features vs Clinicopathologic Features Alone and Genetic Features

	Harrell's concordance index [mean (SD) or median (IQR)]			
Outcome	Clinicopathologic and genetic features	Clinicopathologic features	Genetic features	P values<br>All features test<br>Paired test
Surgery with adjuvant CCRT				
Cancer-specific survival	0.689 (0.050)	0.673 (0.051)	0.556 (0.035)	<0.001 [a]<br>0.022 [c]
Distant metastasis-free survival	0.702 (0.056)	0.688 (0.048)	0.565 (0.032)	<0.001 [a]<br>0.092 [c]
Locoregional recurrence-free survival	0.693 (0.039)	0.678 (0.035)	0.557 (0.030)	<0.001 [a]<br>0.004 [c]
Surgery with adjuvant RT				
Cancer-specific survival	0.700 (0.056)	0.674 (0.054)	0.556 (0.045)	<0.001 [a]<br><0.001 [c]
Distant metastasis-free survival	0.705 (0.079)	0.65 (0.069)	0.605 (0.069)	<0.001 [a]<br><0.001 [c]
Locoregional recurrence-free survival	0.644 (0.064)	0.618 (0.066)	0.543 (0.050)	<0.001 [a]<br>0.004 [c]
Surgery alone [e]				
Cancer-specific survival	0.865 (0.071)	0.888 (0.074)	0.697 (0.107)	<0.001 [b]<br>0.522 [d]

© 2020 Tseng Y-J et al. *JAMA Network Open.*

Distant metastasis-free survival	0.895 (0.087)	0.879 (0.124)	0.768 (0.140)	<0.001 [b]<br>1 [d]
Locoregional recurrence-free survival	0.819 (0.092)	0.827 (0.121)	0.658 (0.111)	<0.001 [b]<br>1 [d]

Abbreviations: CCRT, concurrent chemoradiation; RT, radiotherapy; SD, standard deviation

[a] Repeated ANOVA test, comparing three feature sets; [b] Friedman's test, comparing three feature sets

[c] Pairwise paired t-tests, comparing models using clinicopathologic and genetic features versus those that used clinicopathologic features, alone. P-values are adjusted using the Bonferroni multiple testing correction method.

[d] Pairwise paired Wilcoxon signed-rank test, comparing models using clinicopathologic and genetic features versus clinicopathologic features alone. P-values are adjusted using the Bonferroni multiple testing correction method.

[d] This data point is reported as the median (IQR)

© 2020 Tseng Y-J et al. *JAMA Network Open.*

eTable 4. Features Associated With Prognostic Prediction and the Hazard Ratios

The clinicopathologic and genetic features selected by more than 80% (n≥24) of the elastic-net penalized Cox's proportional hazards regression models in each treatment and survival groups and the hazard ratios. RT: radiotherapy; CCRT: concurrent chemoradiation.

	Cancer-specific survival		Distant metastasis-free survival		Locoregional recurrence-free survival	
Features	Models (%)	HR, mean (SD)	Models (%)	HR, mean (SD)	Models (%)	HR, mean (SD)
Surgery with adjuvant CCRT						
AKT1	87	1.15 (0.10)	-	-	100	1.22 (0.14)
Extranodal extension	100	1.21 (0.11)	100	1.31 (0.14)	100	1.14 (0.05)
FGFR3	-	-	-	-	100	1.12 (0.09)
HRAS	100	1.63 (0.43)	100	2.28 (0.76)	100	1.41 (0.23)
Pathologic N stage	-	-	-	-	100	1.04 (0.01)
Pathologic stage	87	1.08 (0.04)	-	-	100	1.07 (0.03)
Pathologic T stage	100	1.1 (0.05)	-	-	100	1.07 (0.04)
Pathologic tumor invasion depth	100	1.01 (0)	-	-	100	1.01 (0)
Positive lymph nodes on dissection	97	1.02 (0.01)	100	1.04 (0.03)	100	1.03 (0.02)
SMAD4	80	1.24 (0.27)	-	-	100	1.36 (0.39)
Surgery with adjuvant RT						

© 2020 Tseng Y-J et al. *JAMA Network Open.*

AKT1	97	1.21 (0.10)	-	-	90	1.21 (0.08)
BRAF	90	1.11 (0.07)	-	-	-	-
Differentiation	97	1.07 (0.04)	-	-	80	1.07 (0.04)
Extranodal extension	100	1.18 (0.06)	100	1.21 (0.15)	100	1.12 (0.04)
HRAS	100	1.74 (0.30)	100	1.92 (0.64)	100	1.47 (0.21)
Nearest microscopic margin	80	0.99 (0)	-	-	87	0.99 (0)
Pathologic N stage	97	1.03 (0.01)	-	-	100	1.06 (0.02)
Pathologic stage	97	1.09 (0.03)	-	-	83	1.08 (0.04)
Pathologic T stage	100	1.12 (0.04)	-	-	100	1.11 (0.03)
Pathologic tumor invasion depth	100	1.01 (0)	87	1.01 (0.01)	100	1.01 (0)
Positive lymph nodes on dissection	100	1.02 (0.01)	97	1.02 (0.01)	100	1.01 (0)
SMAD4	97	1.25 (0.16)	-	-	97	1.45 (0.23)
Surgery alone						
AKT1	100	1.34 (0.13)	-	-	100	1.37 (0.09)
Differentiation	90	1.09 (0.05)	-	-	97	1.07 (0.04)
Extranodal extension	100	1.22 (0.06)	100	1.26 (0.15)	100	1.15 (0.03)
HNF1A	-	-	-	-	90	0.82 (0.09)
HRAS	100	1.74 (0.31)	100	2.11 (0.67)	100	1.43 (0.17)

© 2020 Tseng Y-J et al. *JAMA Network Open.*

Nearest microscopic margin	-	-	-	-	80	0.99 (0)
Pathologic N stage	-	-	-	-	100	1.06 (0.01)
Pathologic stage	97	1.09 (0.04)	-	-	93	1.07 (0.02)
Pathologic T stage	100	1.12 (0.04)	-	-	100	1.09 (0.02)
Pathologic tumor invasion depth	100	1.01 (0)	-	-	100	1.01 (0)
Positive lymph nodes on dissection	100	1.02 (0.01)	100	1.02 (0.01)	100	1.02 (0)
SMAD4	93	1.27 (0.14)	-	-	100	1.47 (0.19)

© 2020 Tseng Y-J et al. *JAMA Network Open.*

eAppendix. Online Calculator for Survival Risk Stratification in Patients With Advanced Oral Cancer

An online calculator for differentiating risk levels in cancer-specific survival, distant metastasis-free survival, and locoregional recurrence-free survival for postoperative patients with advanced oral cancer. Available at http://shiny.yjtseng.info/content/21/.

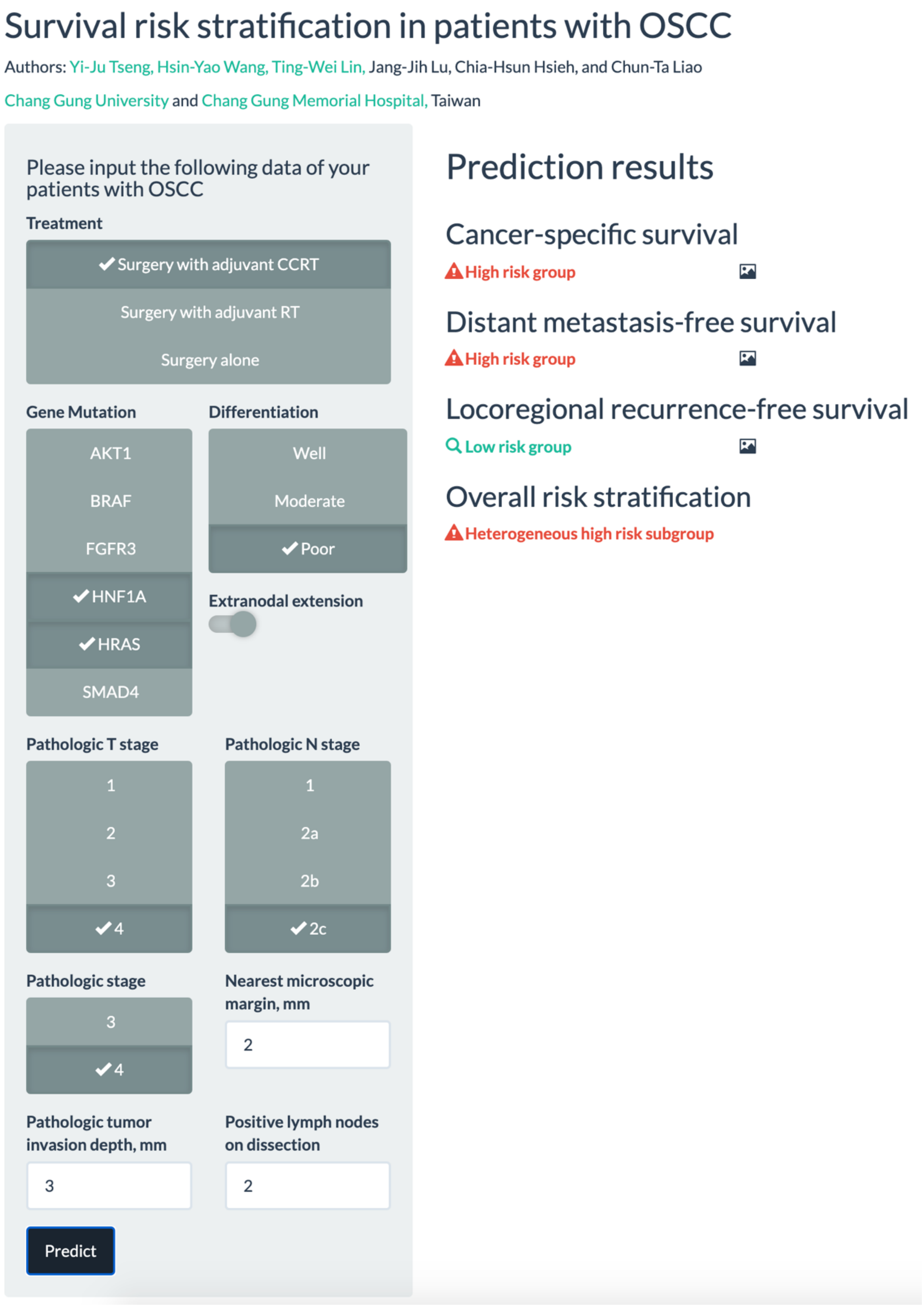

© 2020 Tseng Y-J et al. *JAMA Network Open.*
